# Glycine by enteral route does not improve major clinical outcomes in severe COVID-19: a randomized clinical pilot trial

**DOI:** 10.1038/s41598-024-62321-7

**Published:** 2024-05-21

**Authors:** Mario H. Vargas, Jaime Chávez, Rosangela Del-Razo-Rodríguez, Carolina Muñoz-Perea, Karina Julieta Romo-Domínguez, Renata Báez-Saldaña, Uriel Rumbo-Nava, Selene Guerrero-Zúñiga

**Affiliations:** 1 Departamento de Investigación en Hiperreactividad Bronquial, Instituto Nacional de Enfermedades Respiratorias Ismael Cosío Villegas, Calzada de Tlalpan 4502, CP 14080 Ciudad de México, México; 2 Servicio Clínico de Neumología Pediátrica, Instituto Nacional de Enfermedades Respiratorias Ismael Cosío Villegas, Ciudad de México, México; 3 Servicio de Urgencias, Instituto Nacional de Enfermedades Respiratorias Ismael Cosío Villegas, Ciudad de México, México; 4 Servicio Clínico 3, Instituto Nacional de Enfermedades Respiratorias Ismael Cosío Villegas, Ciudad de México, México; 5 Unidad de Medicina del Sueño, Instituto Nacional de Enfermedades Respiratorias Ismael Cosío Villegas, Ciudad de México, México; 6 Present Address: Servicio de Neumología, Hospital Infantil del Estado de Sonora, Hermosillo, Sonora México

**Keywords:** Viral infection, Respiratory distress syndrome, Randomized controlled trials, Prognostic markers

## Abstract

There is a worrying scarcity of drug options for patients with severe COVID-19. Glycine possesses anti-inflammatory, cytoprotective, endothelium-protective, and platelet-antiaggregant properties, so its use in these patients seems promising. In this open label, controlled clinical trial, inpatients with severe COVID-19 requiring mechanical ventilation randomly received usual care (control group) or usual care plus 0.5 g/kg/day glycine by the enteral route (experimental group). Major outcomes included mortality, time to weaning from mechanical ventilation, total time on mechanical ventilation, and time from study recruitment to death. Secondary outcomes included laboratory tests and serum cytokines. Patients from experimental (n = 33) and control groups (n = 23) did not differ in basal characteristics. There were no differences in mortality (glycine group, 63.6% vs control group, 52.2%, p = 0.60) nor in any other major outcome. Glycine intake was associated with lower fibrinogen levels, either evaluated per week of follow-up (p < 0.05 at weeks 1, 2, and 4) or as weighted mean during the whole hospitalization (608.7 ± 17.7 mg/dl vs control 712.2 ± 25.0 mg/dl, p = 0.001), but did not modify any other laboratory test or cytokine concentration. In summary, in severe COVID-19 glycine was unable to modify major clinical outcomes, serum cytokines or most laboratory tests, but was associated with lower serum fibrinogen concentration.

Registration: ClinicalTrials.gov NCT04443673, 23/06/2020.

## Introduction

Although severity of COVID-19 has become much lower than at the beginning of the pandemic^1^ there is still a need for therapeutic alternatives that benefit sporadic cases who develop severe symptoms. Moreover, the frequent emergence of new strains of SARS-CoV-2 is a latent threat because some may be markedly virulent (the so-called variants of concern), provoking the resurgence of severe COVID-19 cases. The search for therapeutic alternatives is also relevant due to the worrying scarcity of drugs for the treatment of the disease. Current medications only provide a modest beneficial effect on major outcomes, either by containing the virus replication with remdesivir, or by ameliorating the proinflammatory and prothrombotic state with corticosteroids, alone or in combination with the IL-6 receptor blockers tocilizumab or sarilumab, or the JAK pathway inhibitor baricitinib^2^.

In the lungs, SARS-CoV-2 produces cytolytic damage either directly by the virus or indirectly by the release of pro-inflammatory cytokines^3,4^. Severe or critical forms of the disease show important damage of pulmonary vascular endothelium and alveolar epithelial cells, with the production of edema and hyaline membranes, leading to diminished lung function, hypoxemia, and the need for ventilatory support^5^.

During the first waves of the SARS-CoV-2 pandemic, about 20% of COVID-19 patients required monitoring in the hospital setting and an additional 5% required admission to the intensive care unit^6^. Some conditions are known to be risk factors for developing severe forms of the disease, such as advanced age, male sex, systemic hypertension, diabetes, and chronic obstructive pulmonary disease^7,8^, while others increase the risk of death, including systemic hypertension, chronic kidney disease, and cancer^7^. The mechanisms by which these conditions provoke the patient’s worsening are still unclear but, interestingly, many of them are often associated with low serum or plasma levels of glycine^9–15^.

Shortly after the beginning of the COVID-19 pandemic, it was evident that severe cases showed increased serum concentrations of pro-inflammatory cytokines (the so-called “cytokine storm”)^16,17^, including IL-2, IL-7, IL-10, G-CSF, IP-10, MCP1, MIP1A, TNF-α, IP-10, MCP-3, HGF, MIG, and MIP-1α^18,19^. Because such cytokine storm was a pathogenetic mechanism of organ damage, it was suggested that amelioration of this massive cytokine release would be a key therapeutic strategy^20–22^. In line with this proposal, corticosteroids demonstrated a beneficial effect, although moderate, in lowering the mortality among patients with severe COVID-19, and thus their use in this context was recommended by the WHO^23^. However, corticosteroids mainly downregulate some inflammatory cells such as lymphocytes, eosinophils, mast cells, and basophils, but act poorly on other cells such as macrophages (especially in the context of an inflammatory milieu), and neutrophils^24–27^.

Glycine is the simplest amino acid, and besides its structural role in proteins and in the generation of crucial molecules^28^, it is an important inhibitory neurotransmitter in the central nervous system and a chemical mediator in other cell types^29,30^. Glycine activates the glycine receptor (GlyR), which is a ionotropic receptor belonging to the chloride channels family^31^. Once stimulated, this receptor permits that entrance of chloride anions into the cell, causing hyperpolarization of the cell membrane and hence blunting the response to pro-inflammatory stimuli. GlyR is expressed in neurons of the brainstem and spinal cord, but also on adipocytes and inflammatory cells such as alveolar macrophages, Kupffer cells, and neutrophils^32^. Moreover, glycine not only stabilizes the plasma membrane of inflammatory cells but also downregulates the production of superoxide and pro-inflammatory cytokines such as TNF-α and IL-6^33–37^, probably by inhibiting the NF-κB/Iκκ pathway^38^.

In vivo studies in animals corroborate the protective role of glycine in several models of acute insults such as endotoxic shock^39,40^ or ischemia–reperfusion injury^41,42^. In the clinical setting, oral administration of glycine has been used for a long time ago in the management of several ailments^43,44^, and it has been shown that this amino acid lowers glycated hemoglobin and systemic inflammation in patients with type 2 diabetes^45^ and improves the clinical, spirometric and inflammatory status of patients with cystic fibrosis^46^. Importantly, these studies did not find adverse effects of glycine.

In addition, due to the peculiar properties of SARS-CoV-2 for inducing endothelial dysfunction^47^, thrombosis in the micro- and macro-circulation^48^, and cytotoxicity, it becomes particularly relevant that glycine has a cytoprotective effect against different noxious stimuli^49–51^, improves endothelial functioning^52^, and inhibits platelet aggregation^53^. Finally, add-on advantages of glycine are that it is widely available at low cost (~ $25 USD per kg), it is stable at room temperature as a whitish power or fine crystals, it is soluble in water, and it is palatable with a sweetish flavor.

All the above-mentioned properties of glycine provided strong scientific support for exploring its potential usefulness in COVID-19, including its capability to mitigate the SARS-CoV-2-associated cytokine storm as proposed by Li C.Y. in 2020^54^.

Thus, the objective of the present study was to evaluate whether an enteral supplement of glycine could improve major clinical outcomes of inpatients who received mechanical ventilation due to severe COVID-19 (mortality, time to weaning from mechanical ventilation, total time on mechanical ventilation, and time from study recruitment to death). As secondary objectives, we evaluated whether glycine ameliorates the serum cytokine storm and/or modify the elements of routine laboratory analyses.

## Patients and methods

This was a prospective, randomized, open label, controlled clinical trial with two parallel arms carried out from August 2020 to March 2021 at the Instituto Nacional de Enfermedades Respiratorias, a third-level hospital devoted to respiratory diseases located in Mexico City. The protocol was approved by our institutional review board (official name: Comité de Investigación, approval number: C40-20, first registration: 15/06/2020). The trial was conducted in accordance with national regulations and with the principles stated in the Declaration of Helsinki. The authors take full responsibility for the design and conduct of the trial and vouch for the accuracy and completeness of the data. The protocol was registered in ClinicalTrials.gov (NCT04443673, 23/06/2020). Patients of any sex and age with severe COVID-19 were recruited in the emergency room or hospital wards if they fulfilled the following eligibility criteria: (1) there was confirmation of the disease by a PCR test or there was clinical data highly suggestive of severe COVID-19, (2) they were on mechanical ventilation due to the severity of the disease, or the treating medical team considered that they will shortly require mechanical ventilation, (3) they were not participating in another research protocol, and (4) the legal guardian signed an informed consent letter accepting the patient’s participation in the study. Pregnant women were excluded and patients with voluntary discharge were eliminated from the study. The projected sample size was 41 patients per group, postulating a 70% lower mortality in the experimental group from an expected global mortality of 30%. Through a computerized random number generator, patients were allocated either to the experimental group or to the control group.

Participants in the experimental group received a daily enteral supplement of 0.5 g/kg glycine, up to a maximum of 40 g/day. This glycine dose was used by us in a previous study in children with cystic fibrosis^46^, and represents an intermediate dose according to studies in adult populations. For example, in patients with diabetes, Cruz et al.^45^ and Carvajal et al.^55^ administered 15 and 20 g/day, respectively, equivalent to 0.25 and 0.33 g/kg/day in a subject weighing 60 kg. In patients with schizophrenia, Heresco-Levy et al.^56^ employed 0.8 g/kg/day for 6 weeks, while Potkin et al.^57^ and Evins et al.^58^ administered 30 and 60 g/day for 8 weeks, equivalent to 0.5 and 1 g/kg/day, respectively. Both groups (experimental and control) received the usual management according to their critical condition, and there was no restriction about receiving accepted pharmacological approaches for COVID-19 treatment, i.e. corticosteroids, anticoagulants, etc., nor any other medication for comorbidities, but experimental drugs were not allowed. All patients received intragastric enteral nutrition, initiated after 24–48 h of admission and repeated on a daily basis for ~ 18 h/day, except when the patients’ critical condition worsened, for example, during upper gastrointestinal bleeding or septic shock. The energy and protein requirements were calculated by a registered dietitian to provide 20–25 kcal/kg/day and 1.2–2.0 g protein/kg/day through administration of a polymeric formula. The selection of the specific commercial formula was done according to supply availability (Supportan DKN, Pulmocare, Nepro HP, Glucerna, Ensure, etc.).

Glycine (USP grade, powder) was purchased from a local drug store. For its administration, the daily dose was divided in four intakes, each one dissolved in 30–50 ml distilled water and administered through the nasogastric tube while intubated or by the oral route once extubated. Glycine administration was continued until hospital discharge or death.

Results from all laboratory tests solicited by the medical staff during the entire patient’s hospital stay were integrated in a database. Additionally, for protocol purposes, a 5 ml venous blood sample was obtained in plastic tubes (BD Vacutainer Serum or SST) approximately every week to determine serum concentrations of IL-1β, IL-2, IL-4, IL-5, IL-6, IL-7, IL-8, IL-10, IL-12p70, IL-13, IL-17A, granulocyte colony stimulating factor (G-CSF), granulocyte–macrophage colony stimulating factor (GM-CSF), IFN-γ, monocyte chemoattractant protein (MCP)-1, macrophage inflammatory protein (MIP)-1β, and TNF-α (Bio-Plex Human Cytokine 17-Plex, Bio-Rad, TX, USA), by multiplex analysis with a Luminex Bio-Plex 200 (Bio-Rad), and glycine by ELISA (Cloud-Clone Corp., TX, USA). Analytes below the limit of detection were considered to be 0.01 pg/ml lower than the lowest value of its respective reference curve. Due to financial constraints, glycine and cytokine concentrations were only measured in 70% and 79% of patients in the control and glycine groups, respectively.

### Data analysis

#### Impact of glycine on major clinical outcomes

With respect to clinical variables, Fisher’s exact test and Mann–Whitney *U* test were used for group comparison of dichotomic and interval variables, respectively. The latter non-parametric approach was used because some of de interval variables did not follow the normal distribution (Kolmogorov–Smirnov test).

#### Impact of glycine on laboratory results

Due to the highly variable periodicity in which laboratory tests were solicited by the medical staff, they were analyzed using two approaches: (1) by grouping each laboratory parameter in periods of 7 days, starting from the day of recruitment (day 0), and (2) by calculating the weighted mean of each test during the entire hospitalization, with time as the weighting factor, according to the following formula:

$$\overline{x}_{w} = \frac{{\sum {(\overline{x} }_{1,2} \cdot t_{1,2} )}}{T},$$

where $\overline{x }$_1,2_ is the average of any two successive determinations of the laboratory parameter, t_1,2_ is the time elapsed between such two determinations, and T is the total time elapsed between the first and the last determination of the parameter during the whole hospitalization. These two approaches were also applied for the analysis of cytokine concentrations. Some indexes or ratios already reported as having a prognostic factor were also evaluated using the weighted means. Although most of the laboratory parameters achieved the normal distribution (Kolmogorov–Smirnov), individual values were log transformed before their statistical analysis to achieve or improve normality, and their group comparison was assessed by non-paired Student’s *t*-tests.

Statistical analyses were performed in R and Stata v13 programs. Data in the text and illustrations are expressed as mean ± standard error, or the geometric mean and range. Statistical significance was set at two-tails p < 0.05.

### Study protocol amendments

The protocol was first registered in 15/06/2020. An amendment was done on 17/07/2020 to extend the administration of glycine or placebo until the patient’s hospital discharge (the initial plan was the administration of glycine and placebo until the end of the mechanical ventilation or the death), and to allow for the inclusion of patients with up to 48 h of mechanical ventilation (the initial criterium was up to 24 h of mechanical ventilation). This time extension was motivated because of the low number of patients recruited and, in fact, due to the same reason a second amendment was done on 26/08/2020 to allow for the inclusion of patients with any duration of mechanical ventilation.

## Results

**Figure 1:**
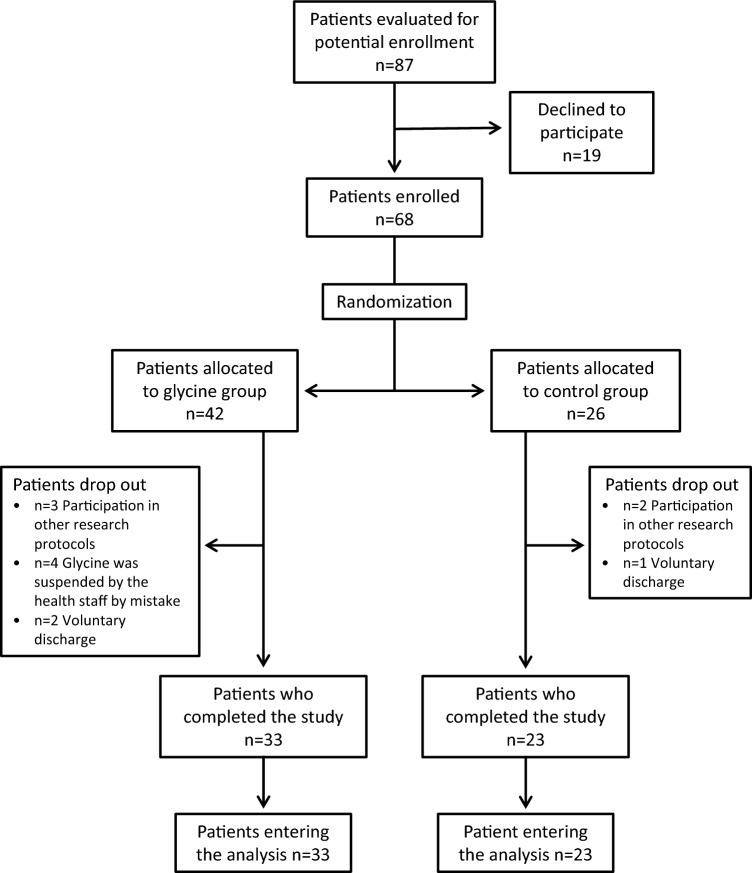
Flow diagram of patients with severe COVID-19.

Although the projected sample size was 82 patients, an interim analysis of the first 68 recruited patients demonstrated no differences in major outcomes between glycine and control groups, so we decided to stop the study early due to futility. The participants’ flow diagram can be seen in Fig. 1. From the 68 patients initially enrolled in the study and randomized, 12 were dropped out due to several reasons (5 were erroneously recruited in another research protocol, 3 had voluntary discharge, and 4 in the glycine group did not receive glycine or it was suspended after few doses by the treating medical staff due to non-medical reasons). Thus, a final population of 56 participants were included in the study, 33 in the glycine group and 23 as controls. All patients had a positive PCR to SARS-CoV-2, except two patients in whom the diagnosis of COVID-19 was based on strong clinical and ancillary grounds, notwithstanding their reiterative negative results of the PCR tests. One patient in the glycine group eventually did not receive mechanical ventilation and was maintained with nasal high-flow oxygen.

**Figure 2:**
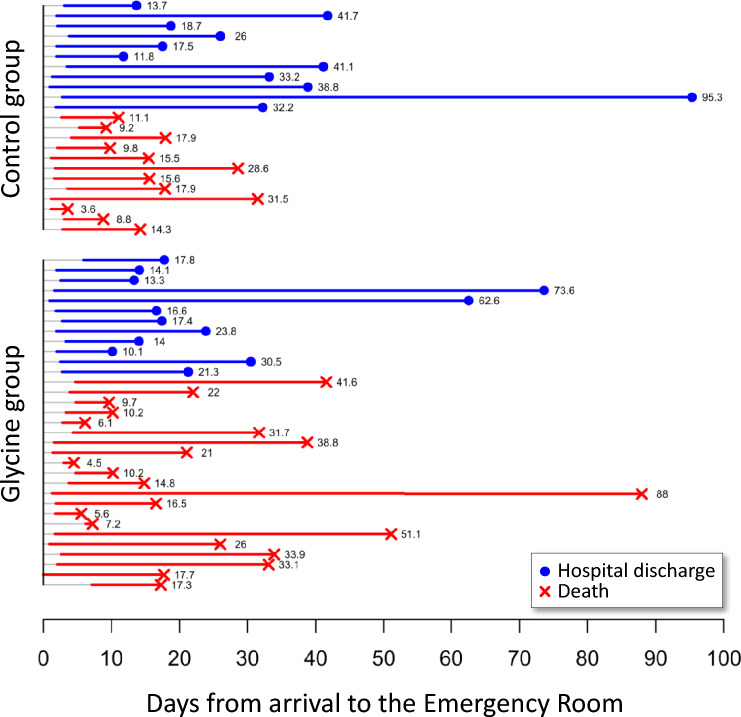
Length of hospital stay of patients in control and glycine groups. Gray lines correspond to the time elapsed between patients’ arrival to the emergency room (day 0) and their recruitment into the study. Solid lines represent the study period of survivors (blue lines) and non-survivors (red lines).

As can be seen in Table 1, patients from both groups did not differ in sex, age, anthropometry, comorbidities, or days elapsed from admission to beginning of mechanical ventilation and/or to recruitment into the study. The length of the hospital stay of each patient is illustrated in Fig. 2.

**Table 1:** Basal characteristics of patients with severe COVID-19 who completed the study.

	Control (n = 23)	Glycine (n = 33)	p*
General data			
Sex, female	5 (21.7)	5 (15.2)	0.74
Age, years	55.2 (31.8 to 86.2)	56 (36.0 to 86.1)	0.52
Weight, kg	80 (64 to 108)	82 (60 to 125)	0.68
Height, cm	167 (150 to 180)	170 (153 to 186)	0.10
BMI, kg/m^2^	30.1 (24.2 to 39.2)	28.7 (22.0 to 46.9)	0.59
Associated conditions			
Hypertension	7 (30.4)	15 (45.5)	0.41
Tobacco habit	3 (13.0)	9 (27.3)	0.33
Diabetes	4 (17.4)	6 (18.2)	1
Cardiovascular disease	2 (8.7)	5 (15.2)	0.69
Alcoholism	2 (8.7)	0 (0)	0.15
Hypothyroidism	1 (4.3)	1 (3.0)	1
Other	3 (13.0)	1 (3.0)	0.29
Study recruitment			
Time from admission to start of mechanical ventilation, days	0.1 (0 to 2.1)	0.2 (− 1.6 to 7.2)^†^	0.53
Time from admission to study recruitment, days	2 (0.9 to 5.3)	2.5 (0 to 7.1)	0.50
Time from start of mechanical ventilation to study recruitment, days	1.8 (− 0.7 to 5.1)	1.8 (− 4.2 to 7.0)^†^	0.62

Data correspond to frequency (percentage) or median (minimum to maximum values).

*Fisher exact test or Mann–Whitney *U* test.

^†^One patient did not receive mechanical ventilation (n = 32).

### Effect of glycine on major clinical outcomes

We did not find statistical differences in major outcomes between control and glycine groups, i.e., they did not differ in mortality, time from recruitment to death, days on mechanical ventilation, and time from study recruitment to end of mechanical ventilation (Table 2). In particular, there was a global mortality of 58.9% among the whole population, and this percentage was rather similar between controls (52.2%) and patients receiving glycine (63.6%, p = 0.60).

**Table 2:** Major clinical outcomes in patients with severe COVID-19 with or without intake of an enteral supplement of glycine.

	Control (n = 23)	Glycine (n = 33)	p*
Mortality	12 (52.2)	21 (63.6)	0.60
Time from study recruitment to death, days	12.7 (2.5 to 30.4)	17.7 (1 to 86.7)	0.35
Total time on mechanical ventilation, days	13.8 (3.5 to 57.1)	14.3 (0 to 87.9)^†^	0.36
Time from study recruitment to end of mechanical ventilation, days	10.6 (6.2 to 55)	9.8 (2.7 to 50.7)	0.95

Data correspond to frequency (percentage) or median (minimum to maximum values).

*Fisher exact test or Mann–Whitney *U* test.

^†^One patient did not receive mechanical ventilation (n = 32).

Although patients who died were slightly older (61.3 ± 12.6 years, mean ± standard deviation) than patients who survived (54.8 ± 14.6 years), in the logistic regression analysis age did not predict survival (p = 0.08), and this lack of influence of age on survival persisted even after group (glycine vs control) and group*age interaction were included as covariables (data not shown).

### Effect of glycine on laboratory tests and serum cytokines

**Figure 3:**
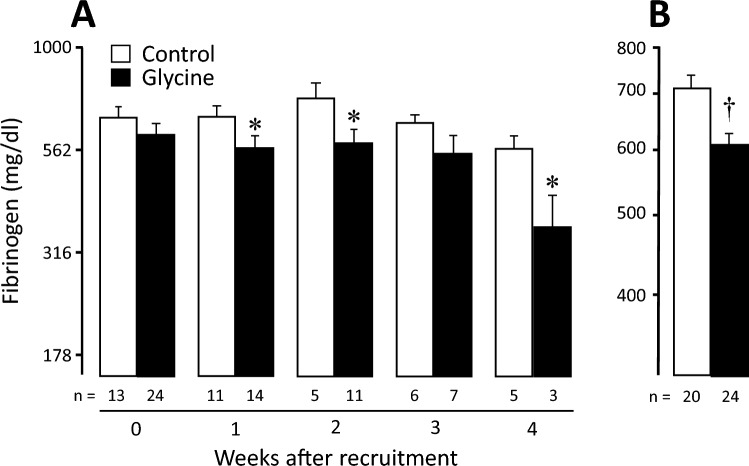
Changes in serum fibrinogen in control and glycine groups. Patients receiving glycine (0.5 g/kg/d) showed lower fibrinogen levels evaluated by week of follow-up (**A**) or by the weighted mean during the whole hospitalization (**B**). The number of patients appears at the bottom of each bar. *p < 0.05 and ^†^p < 0.001.

Individual values of all clinical laboratory parameters and of serum cytokines and glycine, evaluated as the weighted mean during the whole hospitalization, can be observed in the Supplementary Fig. S1. Although many laboratory results were clearly abnormal (e.g. high values of glucose, urea, BUN, neutrophils, GGT, LDH, fibrinogen, D dimer, and ferritin, and low values of calcium, lymphocytes, and albumin), there was no difference between control and experimental groups in most of them. The only laboratory parameter that achieved statistically significant difference between both groups was serum fibrinogen, which was lower in patients receiving glycine, either evaluated per weeks of follow-up (p < 0.05 at weeks 1, 2, and 4, Fig. 3A) or as weighted mean during the whole hospitalization (608.7 ± 17.7 mg/dl vs control 712.2 ± 25.0 mg/dl, p = 0.001, Fig. 3B). The complete set of serum fibrinogen results during the hospital stay of each patient is illustrated in Supplementary Fig. S2). With respect to serum cytokines, seven were undetectable in more than 65% of samples, so they were eliminated from the analysis (IL-1β, IL-2, IL-4, IL-5, IL-12, G-CSF, and GM-CSF). From the remaining cytokines (IL-6, IL-7, IL-8, IL-10, IL-13, IL-17, MCP-1, MIP-1β, TNF-α, IFN-γ), we did not find statistical differences in their serum concentration between groups.

Regarding serum glycine, the first serum sample obtained from day 0 to day 4 of recruitment, which might be viewed as a proxy of the basal glycine status, showed that glycine concentrations ranged from 5.4 to 62.7 µg/ml (geometric mean 12.9 µg/ml) in the control group, and from 1.5 to 42.7 µg/ml (geometric mean 9.5 µg/ml) in the glycine group (p = 0.27) (Supplementary Fig. S3). Finally, the weighted mean of serum glycine during the whole hospitalization was not different between control (20.92 ± 4.11 μg/ml), and glycine groups (14.08 ± 2.27 μg/ml, p = 0.15), which agrees with the variability and the lack of a noticeable pattern of serum glycine concentrations during the hospital stay (Supplementary Fig. S4).

There were no adverse events related to glycine administration.

## Discussion

COVID-19 posed a tremendous challenge for science in the search of therapeutic strategies capable of counteract the ongoing organ damage occurring in severe cases. Until now, besides systemic corticosteroids, few therapeutic options exist, such as remdesivir, tocilizumab, sarilumab and baricitinib, all of which have only a modest beneficial effect^2^. Thus, the need of a better therapeutic approach for severe forms of COVID-19 is still required. In the middle of 2020, we postulated that, due to its characteristic anti-inflammatory and cytoprotective effects, glycine might improve the fate of patients on mechanical ventilation due to severe COVID-19. Unfortunately, results from the present study did not corroborate this expectation inasmuch as glycine administration was unable to modify neither mortality nor other major clinical outcomes, such as time from study recruitment to death, total time on mechanical ventilation, or time from study recruitment to end of mechanical ventilation. Likewise, there was no change in laboratory results or cytokine levels attributable to glycine, except for a glycine-related lower concentration of fibrinogen.

One potential explanation for the null effect of glycine on major outcomes and most clinical laboratory data might be that this amino acid needs a relatively long period of time to fully achieve its anti-inflammatory effect. This possibility is supported by a previous study by our group in which we demonstrated that an 8-week glycine supplementation produced clinical improvement and diminution of some serum and sputum pro-inflammatory cytokines in children with cystic fibrosis^46^. These beneficial effects were more evident at the end of the 8-week period and, due to the cross-over nature of the study, there was evidence of a carry-over effect of glycine, indicating the relatively slow onset of the anti-inflammatory action of glycine. Glycine is widely utilized in many relevant metabolic pathways, including purine (RNA and DNA), heme, glutathione, creatine, serine, and conjugated bile acids biosynthesis^28^, and these requirements are increased in COVID-19 patients, specially in those critically ill^59,60^, which leads to large weight loss^61^. Therefore, it is probably that in the present study glycine preferentially covered these metabolic demands before exerting an inhibitory effect on inflammatory cells. In agreement with this possibility, in the above-mentioned study we found that the serum concentration of glycine slowly rose throughout the 8 week period.

An additional possibility for the lack of effect of glycine was that the critical condition of patients impaired the enteral absorption of glycine^62^ and in this case, perhaps glycine administration by the intravenous route might have been a better approach. Furthermore, because blood concentrations of glycine rapidly decline due to its uptake by tissues^63^ may be a continuous intravenous infusion would have been the best choice. This rapid clearance of glycine from the intravenous compartment, with a half-life as short as < 30 min^64,65^, might also explain why we were unable to demonstrate an increase of serum glycine concentrations in the experimental group, in spite of the enteral administration of this amino acid^66^.

Finally, an additional issue that may have blurred any beneficial effect of glycine is that in these critically ill patients other risk factors insensitive to glycine treatment predisposed them to death.

Among the several biomarkers postulated to be prognostic factors of a worse outcome in severe COVID-19 is fibrinogen. Several studies showed that high levels of fibrinogen and the fibrin degradation product d-dimer are associated with poor prognosis in patients with COVID-19^67^. Thus, the downward trend of fibrinogen concentration in subjects who received glycine may be considered a beneficial effect of this amino acid. Thus, although fibrinogen per se was not related with death, after its adjustment by albumin it was evident that non-survivors had a statistically significant higher fibrinogen/albumin ratio than survivors (p = 0.005, data not shown), and there was a trend for glycine to lower this ratio (one tail p = 0.055).

### Limitations of the study

Due to the stringent conditions in which COVID-19 patients had to be managed, especially during the first waves of the pandemic (strict isolation, limited access to the patients’ room to only those members of the staff strictly necessary for the medical care, unavailability of written medical or nursing records due to potential contagiousness, etc.) it was difficult to carry on a close monitoring of the glycine administration. Thus, although efforts were made to be sure that patients received all glycine doses, we cannot discard that some intakes were omitted. Finally, the present study was carried out during the first waves of the pandemic in Mexico, so it is uncertain whether our results can be applied to other SARS-CoV-2 variants.

## Conclusion

We found that an enteral supplement of glycine was unable to improve major clinical outcomes in patients with severe COVID-19 and did not modify serum cytokines nor most laboratory tests. Nevertheless, glycine was associated with a downward trend of serum fibrinogen concentration. Despite our negative results, the possibility that glycine might exert a beneficial effect if administered by continuous intravenous infusion deserves to be explored.

### Supplementary Information

Supplementary Information.

## Data Availability

Deidentified data base might be available upon reasonable request and in the context of an IRB-approved research project. Requests should be addressed to Mario H. Vargas (mhvargasb@yahoo.com.mx).
