# Supplementary material for: Glycine by enteral route does not improve major clinical outcomes in severe COVID-19: a randomized clinical pilot trial
Source: Sci Rep. 2024 May 21;14:11566. doi: 10.1038/s41598-024-62321-7 (PMC11109244; doi:10.1038/s41598-024-62321-7)
Supplement: Supplementary file 1 — Supplementary Information. [file 41598_2024_62321_MOESM1_ESM.pdf]

# Supplementary material

## Glycine by enteral route does not improve major clinical outcomes in severe COVID-19: a randomized clinical pilot trial

Mario H. Vargas, Jaime Chávez, Rosangela Del-Razo-Rodríguez, Carolina Muñoz-Perea, Karina Julieta Romo-Domínguez, Renata Báez-Saldaña, Uriel Rumbo-Nava, Selene Guerrero-Zúñiga

**Supplementary Figure S1.** Results of laboratory tests and serum concentrations of glycine and cytokines in patients with severe COVID-19.

**Supplementary Figure S2.** Follow-up of serum fibrinogen concentrations in patients with severe COVID-19 included in the study.

**Supplementary Figure S3.** First determination of serum glycine in patients with severe COVID-19 included in the study.

**Supplementary Figure S4.** Follow-up of serum glycine concentrations in patients with severe COVID-19 included in the study.

**Supplementary Table S1.** Plasma or serum glycine concentration in healthy (control) adults.

# Supplementary Figure S1

**Supplementary Figure S1.** Results of laboratory tests and serum concentrations of glycine and cytokines in patients with severe COVID-19. Each circle corresponds to the weighted mean of the variable during the patient's whole hospitalization. Horizontal lines correspond to the upper (red broken line) and lower (blue broken line) limits of normal. The statistical significance (p) for differences between patients receiving usual care plus glycine (0.5 g/k/d) administered by the enteral route or only usual care (control group) was assessed by the Student's t-test.

Abbreviations

**Alk. phosphatase** = alkaline phosphatase; **ALT** = alanine aminotransferase; **AST** = aspartate aminotransferase; **BNP** = brain natriuretic peptide; **BUN/A** = blood urea nitrogen/albumin ratio; **BUN** = blood urea nitrogen; **CPK** = creatine phosphokinase; **CRP/A** = C-reactive protein/albumin ratio; **DD/A** = D-dimer/albumin ratio; **DD/F** = D-dimer/fibrinogen ratio; **ESR** = erythrocyte sedimentation rate; **F/A** = fibrinogen/albumin ratio; **GGT** = gamma-glutamyl transferase; **HDL** = high-density lipoprotein; **IFN** = interferon; **IL** = interleukin; **INR** = international normalized ratio; **L/N** = lymphocyte/neutrophil ratio; **LDH** = lactate dehydrogenase; **LDL** = low-density lipoprotein; **MCH** = mean corpuscular hemoglobin; **MCHC** = mean corpuscular hemoglobin concentration; **MCP** = monocyte chemoattractant protein; **MCV** = mean corpuscular volume; **MIP** = macrophage inflammatory protein; **RDW** = red blood cell distribution width; **TNF** = tumor necrosis factor; **TyG** = triglycerides and glucose index; **VLDL** = very low-density lipoprotein.

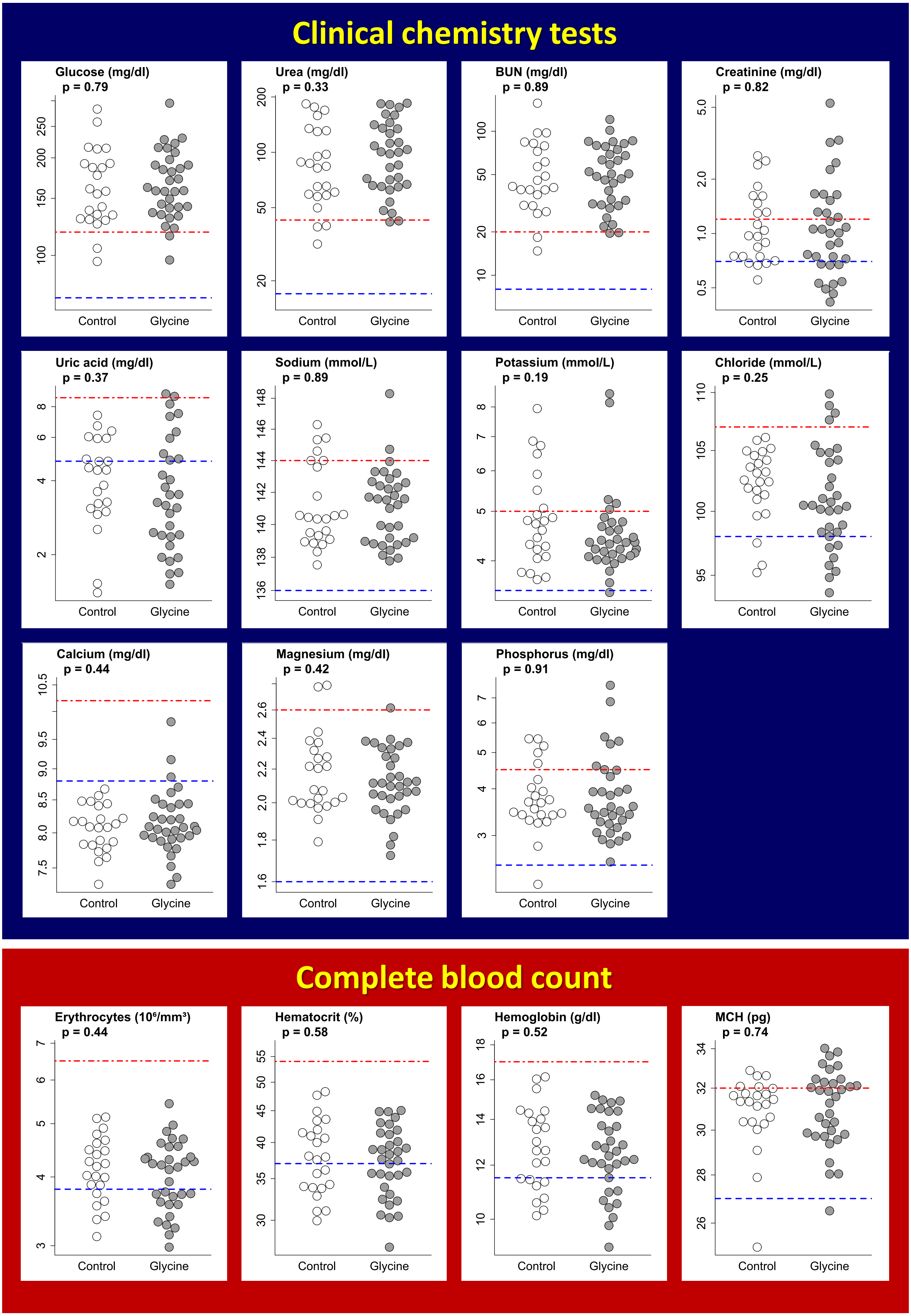


**Supplementary Fig. S1** (see legend in Page 2)

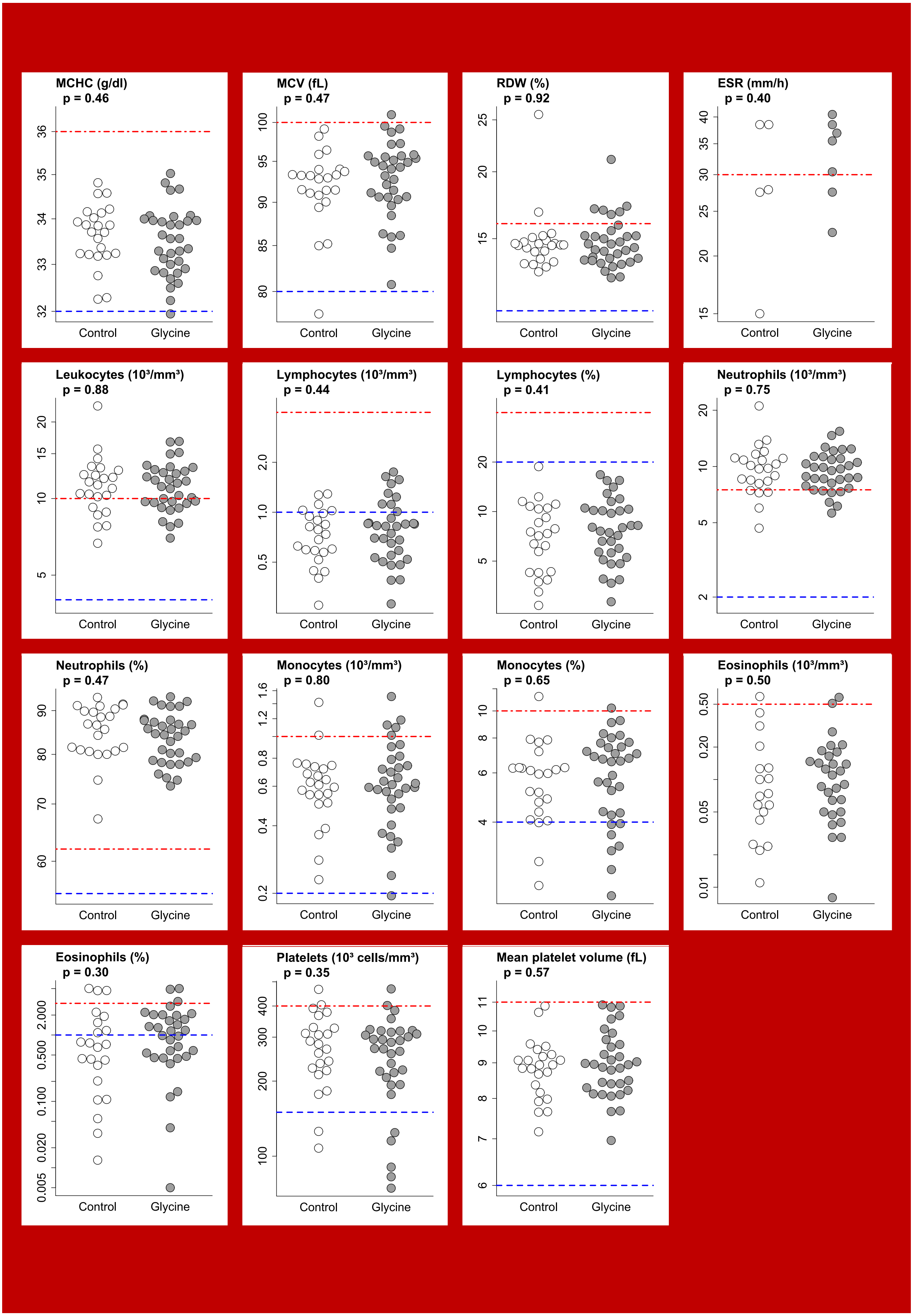


**Supplementary Fig. S1 (continued)** (see legend in Page 2)

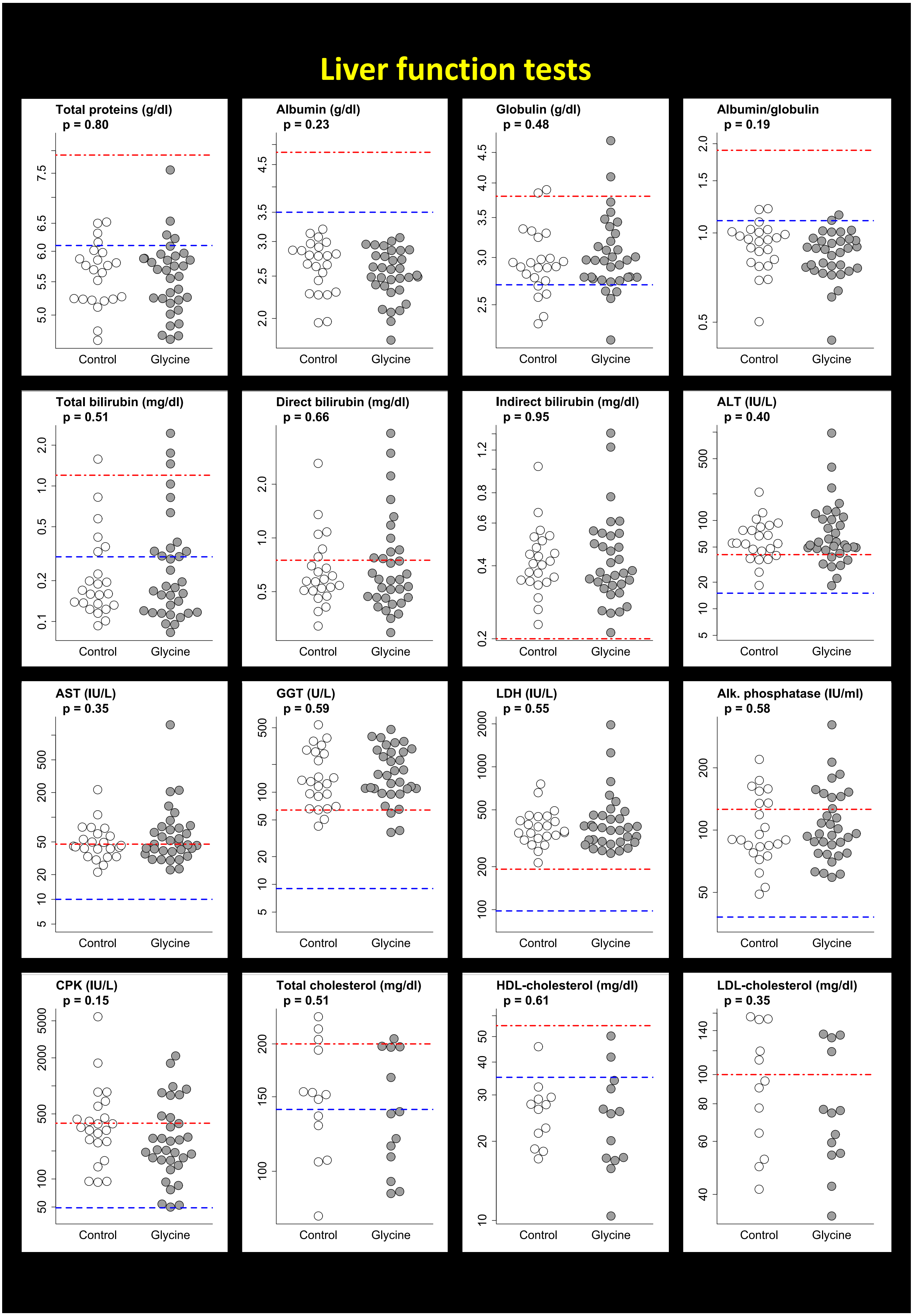


**Supplementary Fig. S1 (continued)** (see legend in Page 2)

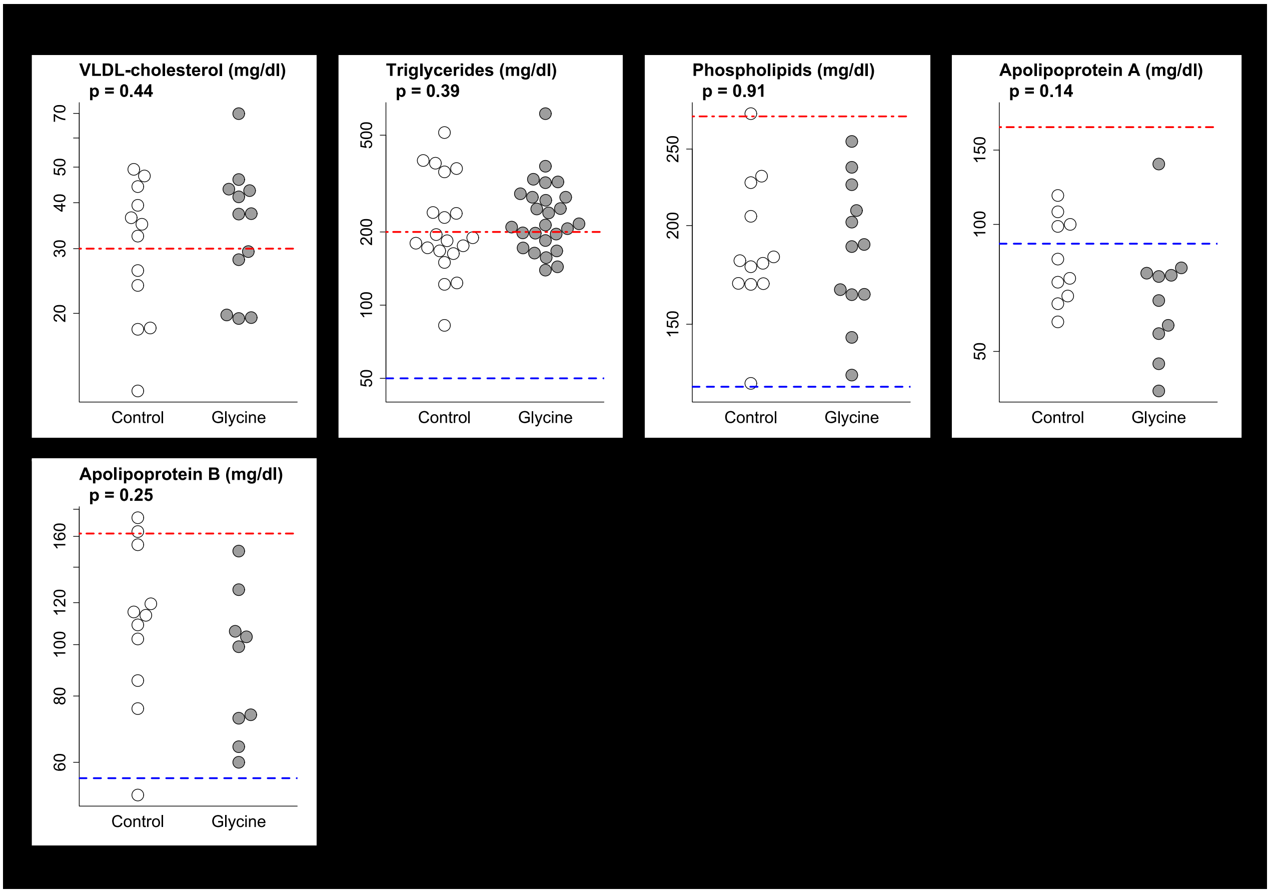


## Coagulation tests

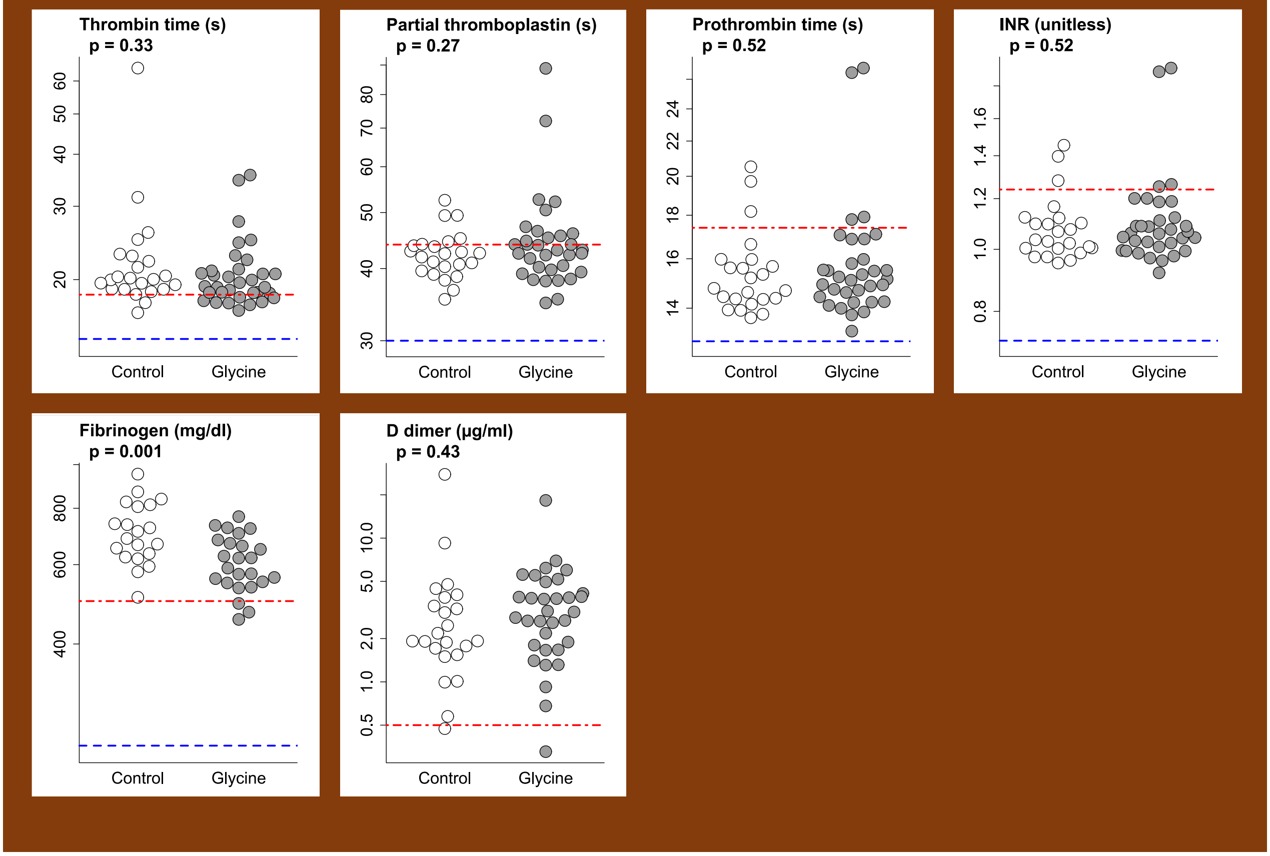


**Supplementary Fig. S1 (continued)** (see legend in Page 2)

# Urianalysis

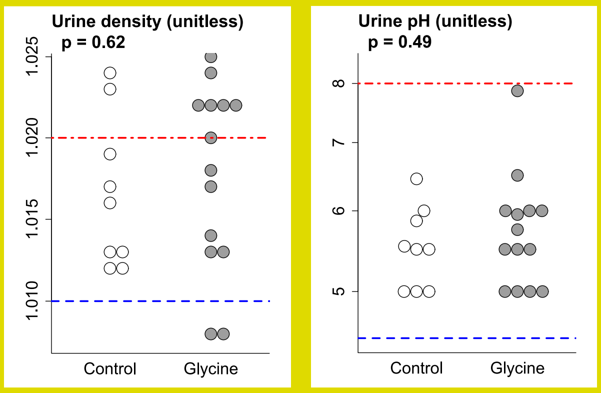


# Serum biomarkers

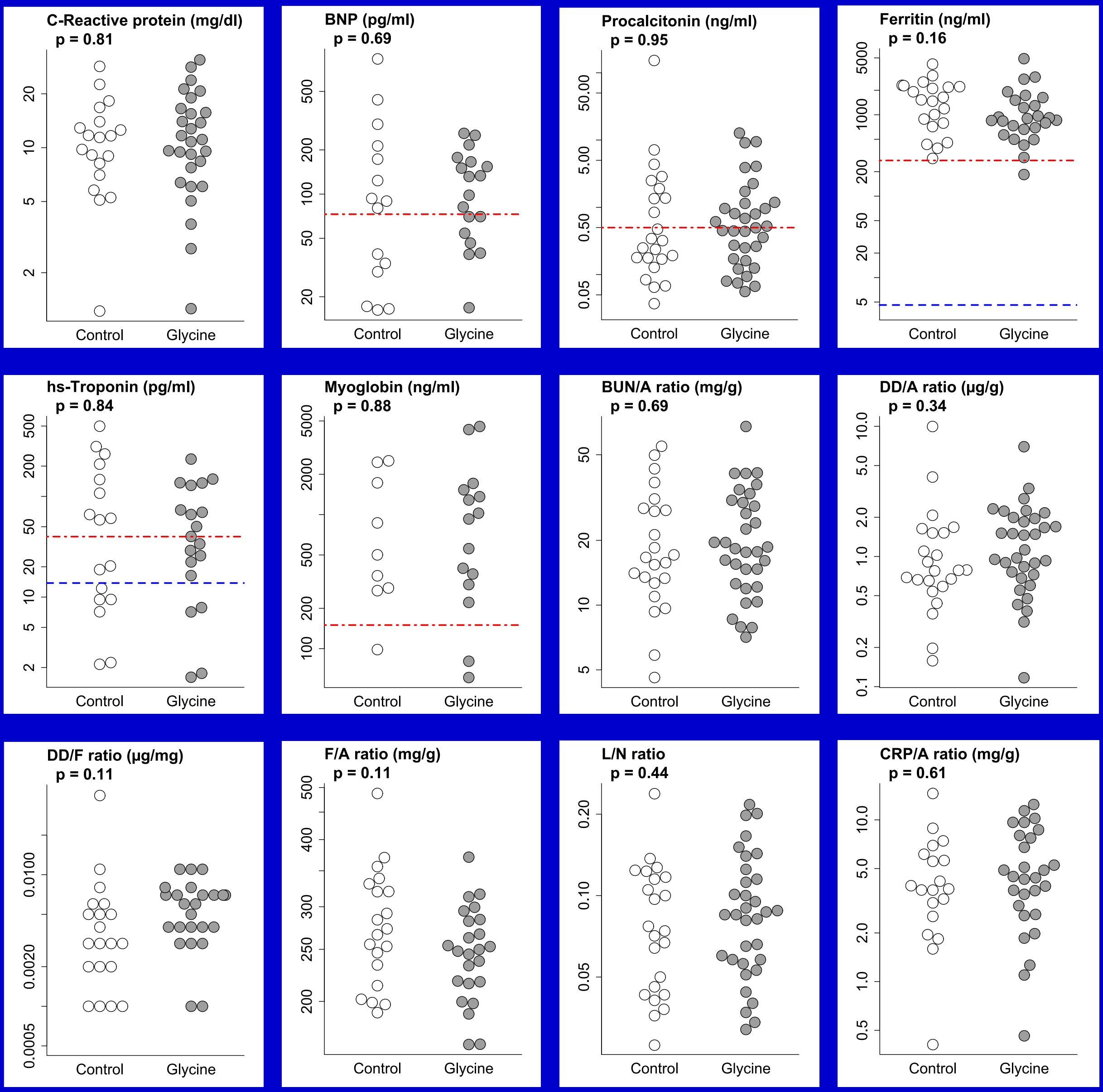


**Supplementary Fig. S1 (continued)** (see legend in Page 2)

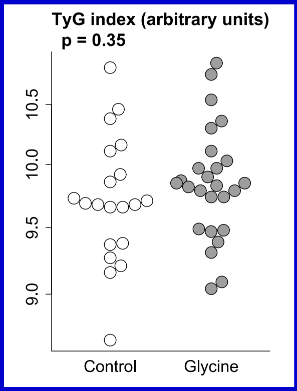


## Serum glycine and cytokines

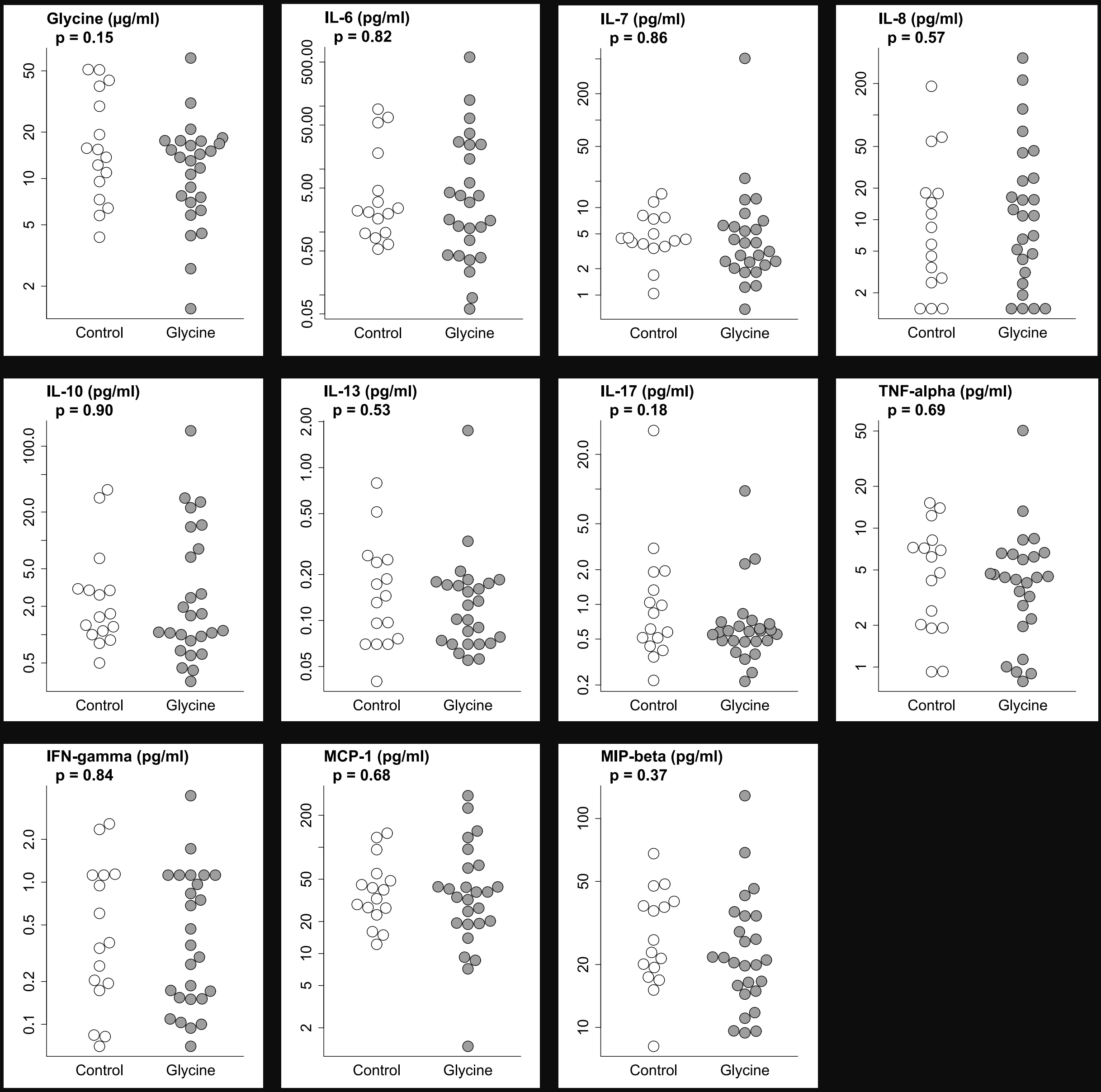


**Supplementary Fig. S1 (continued)** (see legend in Page 2)

# Supplementary Figure S2

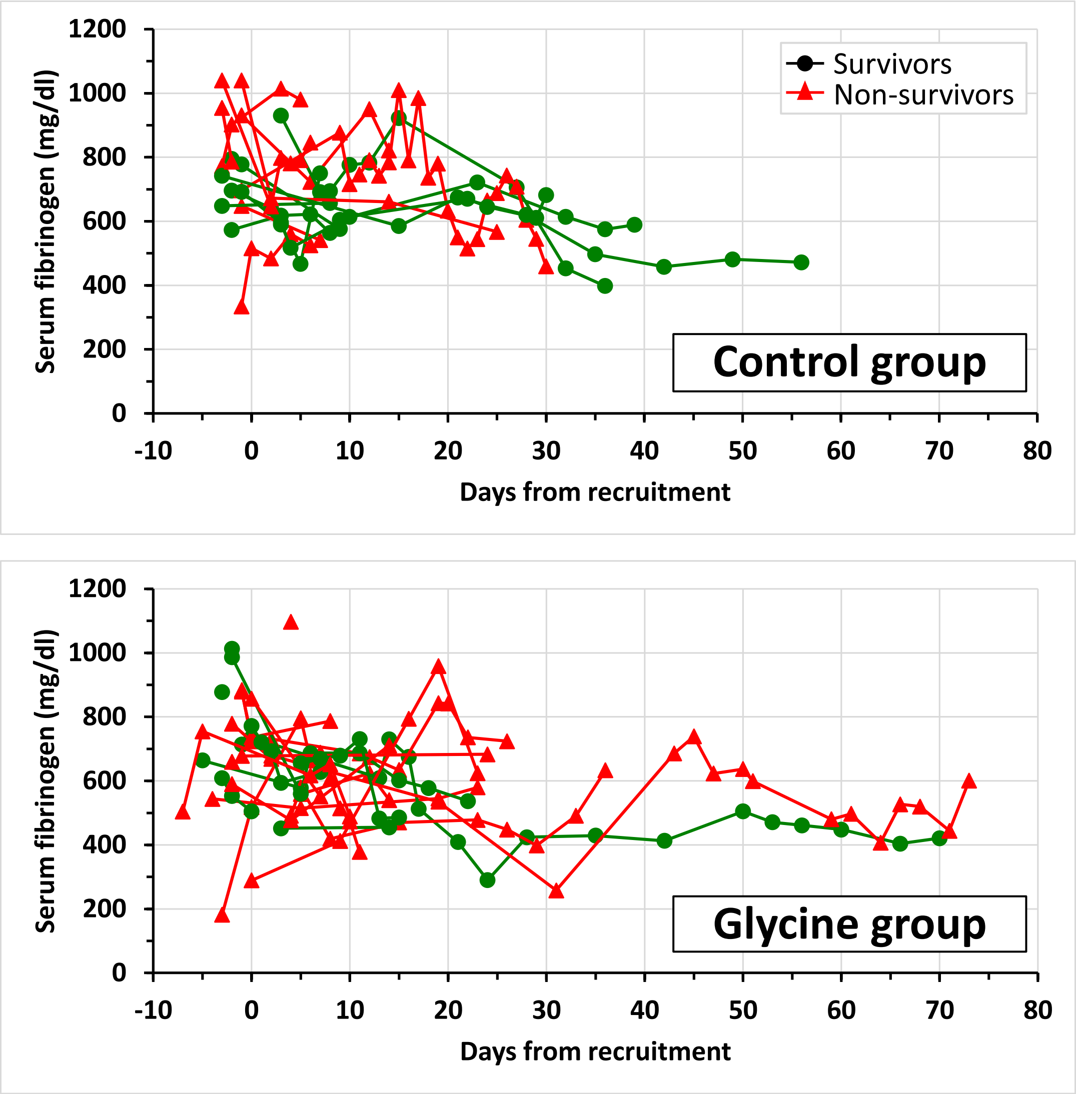


**Supplementary Figure S2.** Follow-up of serum fibrinogen concentrations in patients with severe COVID-19 included in the study. Additional to the usual management, patients in the lower panel received 0.5 g/kg/day glycine by the enteral route. Data corresponds to survivors (–●–) and non-survivors (–▲–).

# Supplementary Figure S3

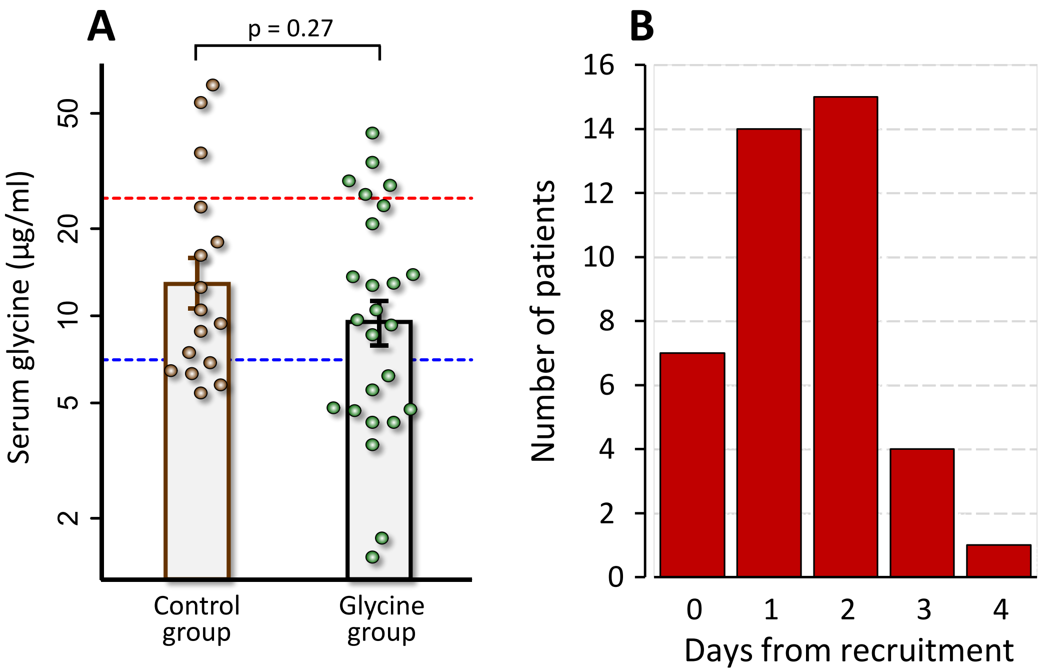


**Supplementary Figure S3.** First determination of serum glycine in patients with severe COVID-19 included in the study. Figures illustrate the glycine concentration (panel A) found in the first serum sample obtained between day 0 and day 4 since recruitment (panel B). Bars in panel A represent the geometric mean ± standard error of the mean. P value was obtained by the non-paired Student's t-test. Horizontal lines correspond to the upper (red broken line) and lower (blue broken line) limits of normal as reported by studies described in the Supplementary Table S1.

# Supplementary Figure S4

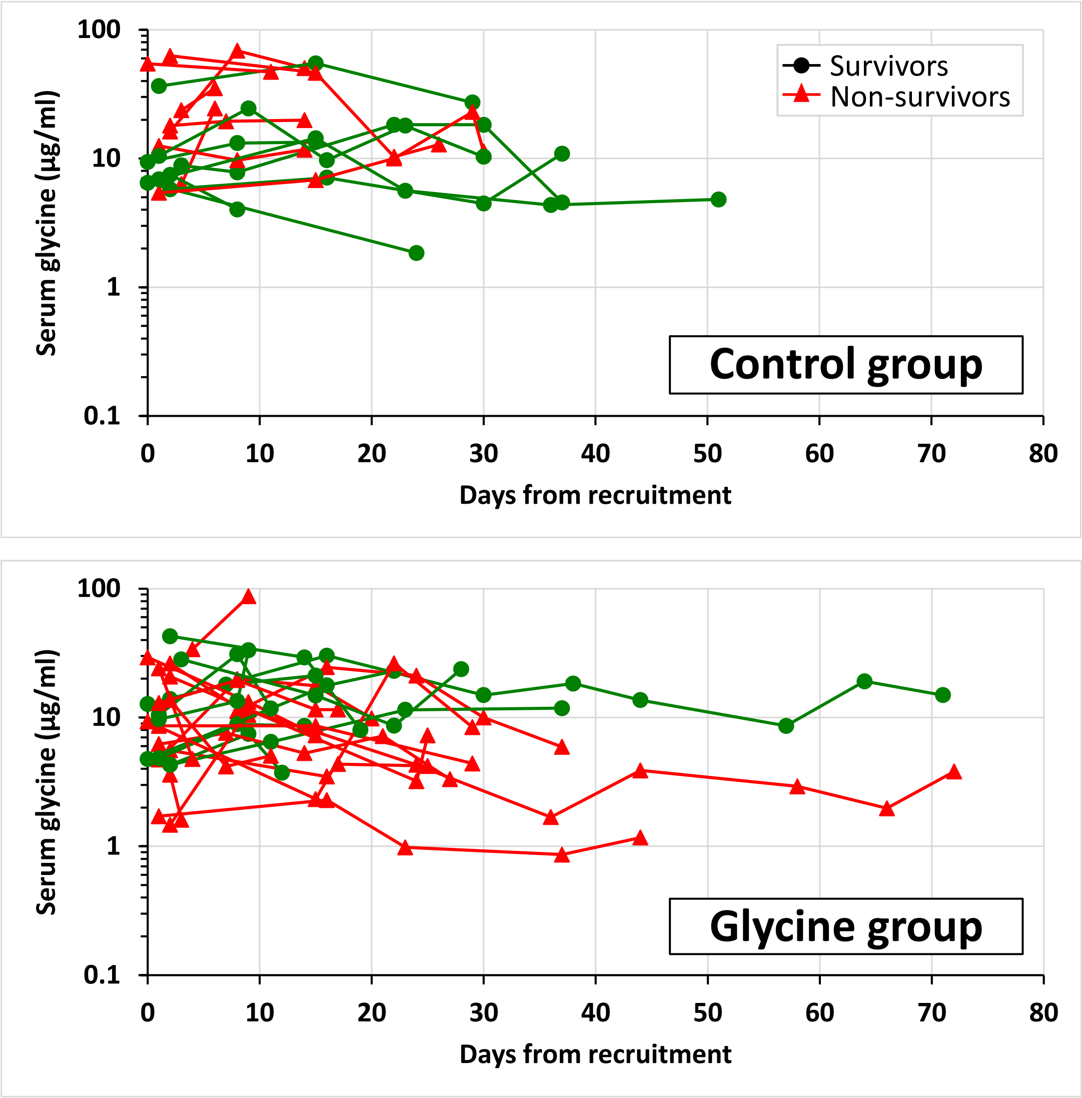


**Supplementary Figure S4.** Follow-up of serum glycine concentrations in patients with severe COVID-19 included in the study. Additional to the usual management, patients in the lower panel received 0.5 g/kg/day glycine by the enteral route. Data corresponds to survivors (-●-) and non-survivors (-▲-).

# Supplementary Table S1

**Supplementary Table S1.** Plasma or serum glycine concentration in healthy (control) adults.

| | | | Glycine (μg/ml) | | | |
|---|---|---|---|---|---|---|
| **Authors, year** | **Country** | **Population** | **Mean** | **SD** | **LLN** | **ULN** |
| Iguacel et al., 2021 | Europe | 3768 | 15.2 | 5.1 | **5.1** | **25.3** |
| Yamamoto et al., 2016 | Japan | 1889 | | | **11.3** | **27.8** |
| Armstrong et al., 1973 | USA | 194 | 20.3 | 6.0 | **8.5** | **32.1** |
| Scriver et al., 1985 | Canada | 80 | 17.4 | 3.3 | **10.9** | **23.9** |
| Divino-Filho et al., 1997 | Germany | 27 | 18.6 | 5.0 | **8.9** | **28.3** |
| Klassen et al., 2001 | Guatemala | 22 | 15.8 | 6.0 | **4.0** | **27.5** |
| Stegink et al., 1991 | USA | 12 | 18.0 | 4.0 | **10.2** | **25.9** |
| Basun et al., 1990 | Sweden | 11 | 16.9 | 4.8 | **7.5** | **26.3** |
| | | **Weighted mean according to population size =** | | | **7.04** | **25.44** |

*LLN=Lower limit of normal; ULN=upper limit of normal*

**References**


Armstrong, M. D. & Stave, U. A study of plasma free amino acid levels. II. Normal values for children and adults. Metabolism: clinical and experimental 22, 561-569, doi:10.1016/0026-0495(73)90069-3 (1973).

Basun, H. et al. Amino acid concentrations in cerebrospinal fluid and plasma in Alzheimer's disease and healthy control subjects. Journal of neural transmission. Parkinson's disease and dementia section 2, 295-304, doi:10.1007/bf02252924 (1990).

Divino-Filho, J. C., Barany, P., Stehle, P., Furst, P. & Bergstrom, J. Free amino-acid levels simultaneously collected in plasma, muscle, and erythrocytes of uraemic patients. Nephrol Dial Transplant 12, 2339-2348, doi:10.1093/ndt/12.11.2339 (1997).

Iguacel, I. et al. Associations between dietary amino acid intakes and blood concentration levels. Clin Nutr 40, 3772-3779, doi:10.1016/j.clnu.2021.04.036 (2021).

Klassen, P., Fürst, P., Schulz, C., Mazariegos, M. & Solomons, N. W. Plasma free amino acid concentrations in healthy Guatemalan adults and in patients with classic dengue. Am J Clin Nutr 73, 647-652, doi:10.1093/ajcn/73.3.647 (2001).

Scriver, C. R., Gregory, D. M., Sovetts, D. & Tissenbaum, G. Normal plasma free amino acid values in adults: the influence of some common physiological variables. Metabolism: clinical and experimental 34, 868-873, doi:10.1016/0026-0495(85)90112-x (1985).

Stegink, L. D. et al. Plasma amino acid concentrations and amino acid ratios in normal adults and adults heterozygous for phenylketonuria ingesting a hamburger and milk shake meal. Am J Clin Nutr 53, 670-675, doi:Plasma amino acid patterns in normal Thais and in patients with chronic renal failure (1991).

Yamamoto, H. et al. Reference intervals for plasma-free amino acid in a Japanese population. Annals of clinical biochemistry 53, 357-364, doi:10.1177/0004563215583360 (2016).
